# Morphological, physiological, cytological and phytochemical studies in diploid and colchicine-induced tetraploid plants of *Fagopyrum tataricum* (L.) Gaertn

**DOI:** 10.1186/s40529-016-0157-3

**Published:** 2017-01-02

**Authors:** Lin-Jiao Wang, Mao-Yin Sheng, Pei-Cai Wen, Jia-Ying Du

**Affiliations:** 1 Karst Research Institute, Guizhou Normal University, Baoshanbei Road 180, Guiyang, 550001 P. R. China; 2 School of Life Science, Nanjing University, Hankou Road 32, Nanjing, 210093 P. R. China; 3 National Engineering Research Center for Karst Rocky Desertification Control, Guizhou Normal University, Baoshanbei Road 180, Guiyang, 550001 P. R. China; 4 State Key Laboratory Incubation Base for Karst Mountain Ecology Environment of Guizhou Province, Baoshaobei Road 180, Guiyang, 550001 P. R. China

**Keywords:** *Fagopyrum tataricum*, Colchicine, Tetraploid, Chromosome counting, Morphological characteristics

## Abstract

**Background:**

Tartary buckwheat are very popular as an important functional food material and its cultivation is very widespread in our whole world, but there obviously lack works in the researches of genetic breeding for agricultural and medicinal utilization. The aim of this study is to obtain good germplasm resources for agricultural and medicinal use of tartary buckwheat (*Fagopyrum tataricum*) by inducing the tetraploid plants.

**Results:**

Four cultivars of *F. tataricum*, that is, Qianwei 2#, Jinku 2#, Chuanqiao 1#, and Liuqiao 1# were selected to experiment. The tips of seedlings with two true leaves were treated by 0.25% (w/v) colchicine solution for 48, 72, and 96 h, respectively. The chromosome number of treated plants was determined by metaphase chromosome counting of root tip cells and PMCs (pollen mother cells) meiosis observation. Tetraploid induction successfully occurred in all three treatments with an efficiency ranging from 12.13 to 54.55%. The chromosome number of the diploid plants was 2*n* = 2*x* = 16, and that of the induced tetraploid plants was 2*n* = 4*x* = 32. The typical morphological and physiological qualities were compared between the control diploid and corresponding induced tetraploid plants. Results showed that the induced tetraploid plants had obviously larger leaves, flowers, and seeds. Moreover, the content of seed protein and flavonoid were also increased in the tetraploid plants. The pollen diameter and capsule size of diploid plants were significantly smaller than those of tetraploid plants.

**Conclusion:**

*Fagopyrum tataricum* can be effectively induced into tetraploids by colchicines. The tetraploid induction can produce valuable germplasm resources for breeding and is a practicable breeding way in *F. tataricum*.

## Background

Buckwheat as an important, highly nutritional nonpoaceous crop has great potential as source of food, forage, and medicine (Lin 1994; Holasova et al. 2002; Tang and Wang 2010; Pan and Chen 2010). Buckwheat originates from China and is widely distributed in Asia and Europe (Hou et al. 2016). The tartary buckwheat (*Fagopyrum tataricum* (L.) Gaertn), a diploid species (2n = 2x = 16), belongs to the big-achene group of genus *Fagopyrum* (Chen et al. 2004; Sheng et al. 2011). Tartary buckwheat grain, as an important functional food material, contains proteins with high biological value and balanced amino acid composition; relatively high crude fiber and vitamins B1, B2, and B6; and more rutin than common buckwheat (*F. esculentum* Moench) (Pan and Chen 2010; Wang et al. 2015). The products of tartary buckwheat are very popular and its cultivation is widespread in whole world (Lin 1994). Although lots of cultivation and utilization have been done in tartary buckwheat, there obviously lack studies in the genetic breeding of tartary buckwheat for agricultural and medicinal utilization (Ohnishi and Ohta 1987; Chen et al. 2007).

The polyploidy induction is a significant method for the production of new germplasm resources applicable for plant genetic breeding (Tang et al. 2010). Polyploidy induction has been used as a breeding method in many plants, especially, in crop, horticulture and medical botany. Induced polyploidy can enhance qualities for agriculture, medicine and horticulture utilization (Tang et al. 2010) and improve resistibility to environmental stresses and diseases (Ahloowalia 1967). Besides, polyploidy induction can lead to content change of secondary metabolites (Majdi et al. 2010). Colchicine has been found to have a significant effect on polyploid induction and is widely used for inducing polyploidy in plants (Tang et al. 2010) because it can effectively arrests mitosis at the anaphase stage (Kunitake et al. 1998; Kermani et al. 2003). Until now, numerous researchers reported that germplasm resources have been innovated using polyploidization techniques in many crop or medicinal plants (Ahloowalia 1967; Kermani et al. 2003; Tang et al. 2010; Ye et al. 2010; Kanoktip et al. 2014) including *F. esculentum* (Zhu and Gao 1988; Lian and Chen 2013). Limited works regarding tartary buckwheat, polyploidy induction works have been reported (Hou et al. 2002; Zhou et al. 2012). In the present study, the tetraploid *F. tataricum* plants were successfully induced by colchicine. The chromosome number of the induced plants was confirmed by cytological identification, and the morphological features of the induced tetraploid plants were recorded. The germplasm characteristics and breeding utilization of such tetraploid materials were discussed. The aim of this present study was to produce novel and valuable tetraploid germplasm resources for the utilization of agriculture and medicine in *F. tataricum*.

## Methods

### Plant material

Mature seeds of the following four *F. tataricum* cultivars (2*n* = 2*x* = 16): Qianwei 2#, Jinku 2#, Chuanqiao 1#, and Liuqiao 1#, were collected from Guizhou, Shanxi, Sichuan, and Yunnan province of China, respectively. Before experiment in this study, they were stored in a freezer at temperature of 0–2 °C. The field testing was conducted during 2012–2013 year in the experimental field site of Guizhou Normal University, Guiyang, China. The laboratory test was conducted in the State Key Laboratory Incubation Base for Karst Mountain Ecology Environment of Guizhou Province, Guiyang, China.

### Tetraploid induction

Before germination, seeds, firstly, were disinfected by 0.3% K_2_MnO_4_ for 20 min, then washed with distilled water for three times and dried at a loft drier with 25 °C for about 24 h and finally sown into flower pots. The pots were covered with transparent plastic film and incubated under greenhouse conditions. Colchicine was applied to the apical meristem of seedlings in the stage of two true leaves. Three treatment durations (i.e. 48, 72 and 96 h) were studied to determine the optimum colchicine exposure times at concentration of 0.25% (w/v) for tetraploid induction. Absorbent cotton balls sucked with a solution of colchicines (0.25%, w/v) were wrapped around the apical tips of seedlings when two true leaves of the emerged seedlings appeared. The experiments were conducted in a greenhouse and continued for the three treatment durations (i.e. 48, 72 and 96 h); the colchicines solution was dropped to the absorbent cotton balls twice a day, at 8:30 and 17:30 o'clock. After the treatment durations, the absorbent cotton balls were removed from the seedling tips, and then the seedling tips were cleaned by distilled water and nursed further in the greenhouse. 50 seedlings were experimented for each treatment. After 2 weeks of the durations of colchicines treatment, death rate on young plants was recorded.

### Chromosome counting and meiosis observation

The ploidy levels were determined by chromosome counting of root tip cells, and confirmed by PMCs (pollen mother cells) meiosis observation. Before induction of polyploidy, the chromosome number was determined in root tips of germinated seeds. For the induced plants, seeds were obtained by self-pollination of each treated plant and the chromosome counting was also conducted in root tips. When root tips grew to 1–2 cm long, root rips were cut and pretreated by the method of soaking in a saturated solution of α-bromine naphthalin at room temperature for 5 h. After pretreated, root tips were washed by distilled water for three times and then immersed in Carnoy’s liquid (ethanol:acetic acid = 3:1) for 12–20 h. After three rinses by distilled water, the root tips were soaked in 1 N HCl at 60 °C for 8 min and then moved to distilled water. After that, the root tips were stained by improved Carbolfuchsin, and squashed for chromosome observation. For meiotic investigation, PMCs in young flowers of about one week age were fixed in Carnoy II solution and stained in 2% (w/v) aceto-carmine solution. Images of mitotic chromosomes in the root tip and meiosis were photo by a DP70 digital camera in a BX51 microscope (Olympus, Japan).

### Morphological and physiological characteristics determinations

The morphological and physiological characteristics were compared between the control diploid plants and corresponding induced tetraploid plants. Parameters of plant height, plant breadth, leaf length, leaf width, inflorescence length and flower size were directly measured by a ruler. A digital caliper was used to measure the characteristics of leaf thickness and diameter of basal stem. Pollen grains were collected from flowers at anthesis, fixed in Carnoy II solution overnight, and then stained by 1% (v/v) I_2_-KI solution to detect pollen vitality. The pollen diameter (average of 30 pollens) was measured by a light microscope (magnification of 40×). Seed length was measured by a digital caliper and weight of 1000 seeds was determined by a balance scale.

### Determination of seed protein and flavonoid content

The seed protein and flavonoid content was determined and compared between the control diploid plants and corresponding induced tetraploid plants. Mature seeds were harvested, dried to constant weight, and then ground to a fine powder for the determination of seed protein and flavonoid content. An automatic micro-Kjeldahl procedure was used to determine seed protein content using a conversion factor of 6.25 times the N content. Seed flavonoid content of each sample was determined following colorimetric method (Chang et al. 2002). Briefly, 1 mL solution of each plant extracts (at 0.4% w/v) in methanol were separately mixed with 4 mL of methanol, 2 mL of 10% aluminium chloride, and 3 mL of 1 M potassium acetate, and left at room temperature for 30 min. The absorbance of the reaction mixture was measured at 420 nm with a double beam Perkin Elmer UV/Visible spectrophotometer (USA). The calibration curve was prepared by preparing rutin solution at concentrations 1.25–20 μg ml^−1^ in methanol.

### Statistical analysis

Comparisons of quantitative data between two variable groups were made using Student’s unpaired *t* test (two-sample *t* test), and morphological variation rates among >2 groups were analysed using ANOVA and multiply comparison among levels of each factor and among combinations were analysed using Duncan’s test (Sheng et al. 2011). All statistical analyses were performed by SPSS 16.0 software (Du 1999).

## Results

### Tetraploid induction

The effects of different durations (48, 72, and 96 h) with 0.25% (w/v) colchicines solution on death rate and induction efficiency of tetraploid induction in four chosen cultivars of *F. tataricum*, were examined and recorded in Table 1. Results showed that the solution of colchicines was detrimental to growth and survival of seedlings. The growth of untreated plants (control) was faster than the plants treated with colchicines solution. The death rate (%) and tetraploid induction efficiency (%) were obvious differences among three different durations of colchicines treatment (Table 1). All the four cultivars can be induced tetraploids successfully, but the induction efficiency were obvious different. Among the twelve treatment combinations, the tetraploid induction efficiency ranged from 12.13 to 54.55%. Multi-comparison analysis indicated that the induction efficiency of the treatments of Qianwei 2# and Chuanqiao 1# with the treatment duration of 72 h are the highest (Table 1). The mortality rates of treated seedling ranged from 2 to 10%, and along with the treatment duration increase, the mortality rates become higher.

**Table 1:** Effects of colchicines (0.25%) treated duration on death rate, and tetraploid induction efficiency in treated cultivars of *Fagopyrum tataricum*

Cultivar	Duration of treatment (h)	Number of seedlings	Death rate (%)	Tetraploid induction efficiency (%)
Qianwei 2#	48	50	2.00	29.15 D
	72	50	6.00	53.23 A
	96	50	10.00	41.45 BC
Jinku 2#	48	50	2.00	12.13 E
	72	50	4.00	31.24 D
	96	50	6.00	19.35 E
Chuanqiao 1#	48	50	4.00	50.34 B
	72	50	6.00	54.55 A
	96	50	8.00	48.56 B
Liuqiao 1#	48	50	4.00	18.34 E
	72	50	8.00	30.45 D
	96	50	10.00	20.04 E

Death rate was recorded after 2 weeks of colchicines treatment

*Different capital letters* indicate significance of the difference between two mean values at the *p* = 0.01 level, as tested by Duncan’s test

### Cytological differences

**Fig. 1:**
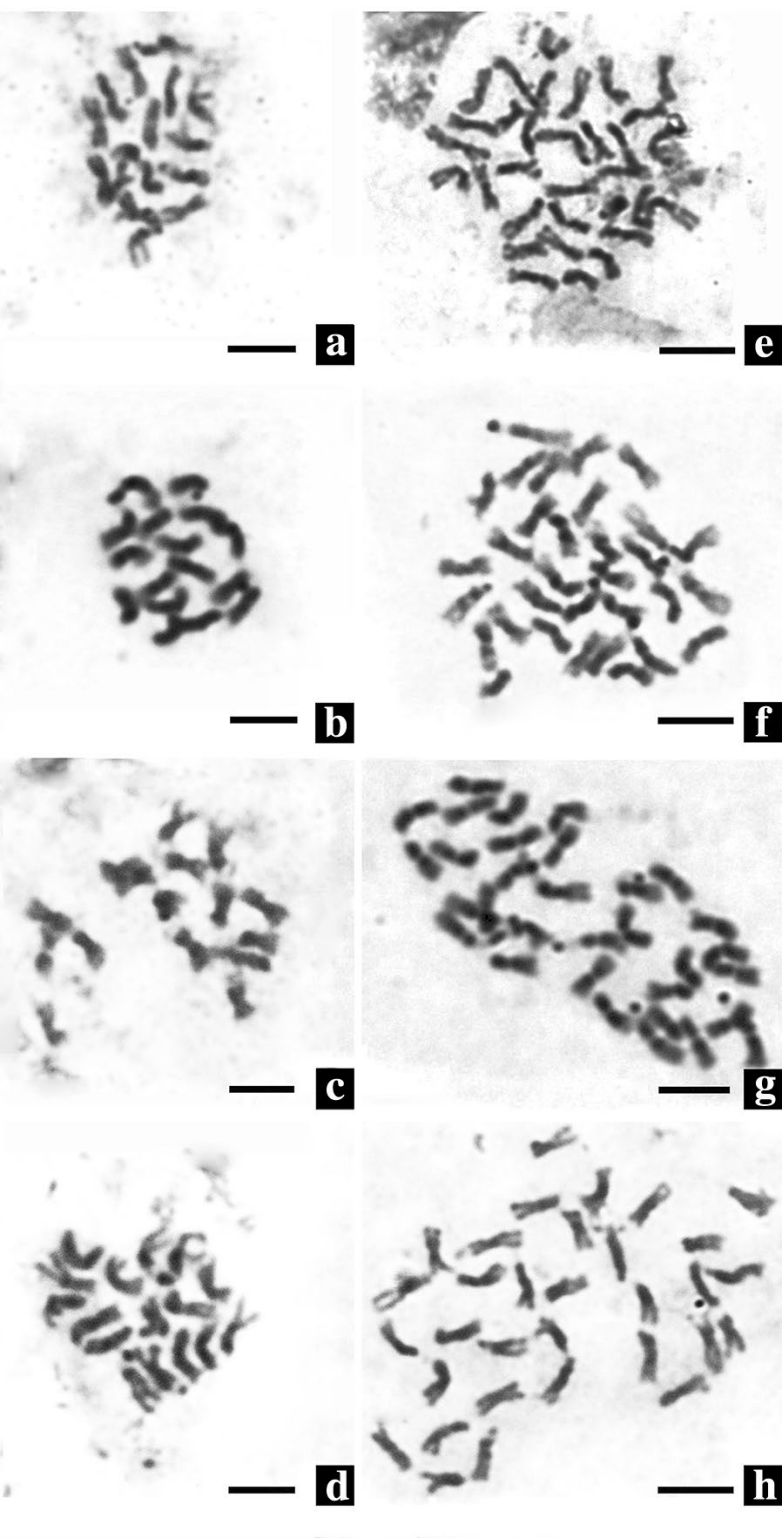
Chromosomes of root tip cell of the diploid *F. tataricum* cultivars (**a**–**d**) and their corresponding induced tetraploids (**e**–**h**) (**a**/**e** Qianwei 2#; **b**/**f** Jinku 2#; **c/g** Chuanqiao 1#; **d**/**h** Liuqiao 1#). *Bars* 5 μm

**Fig. 2:**
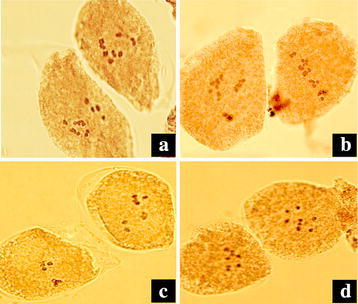
Chromosome configuration of meiotic MI stage of the diploid and induced tetraploid *F. tataricum* (**a** 4*x* = 8IV, **b** 4*x* = 4IV + 8II, **c** and **d** 2*x* = 8II)

**Fig. 3:**
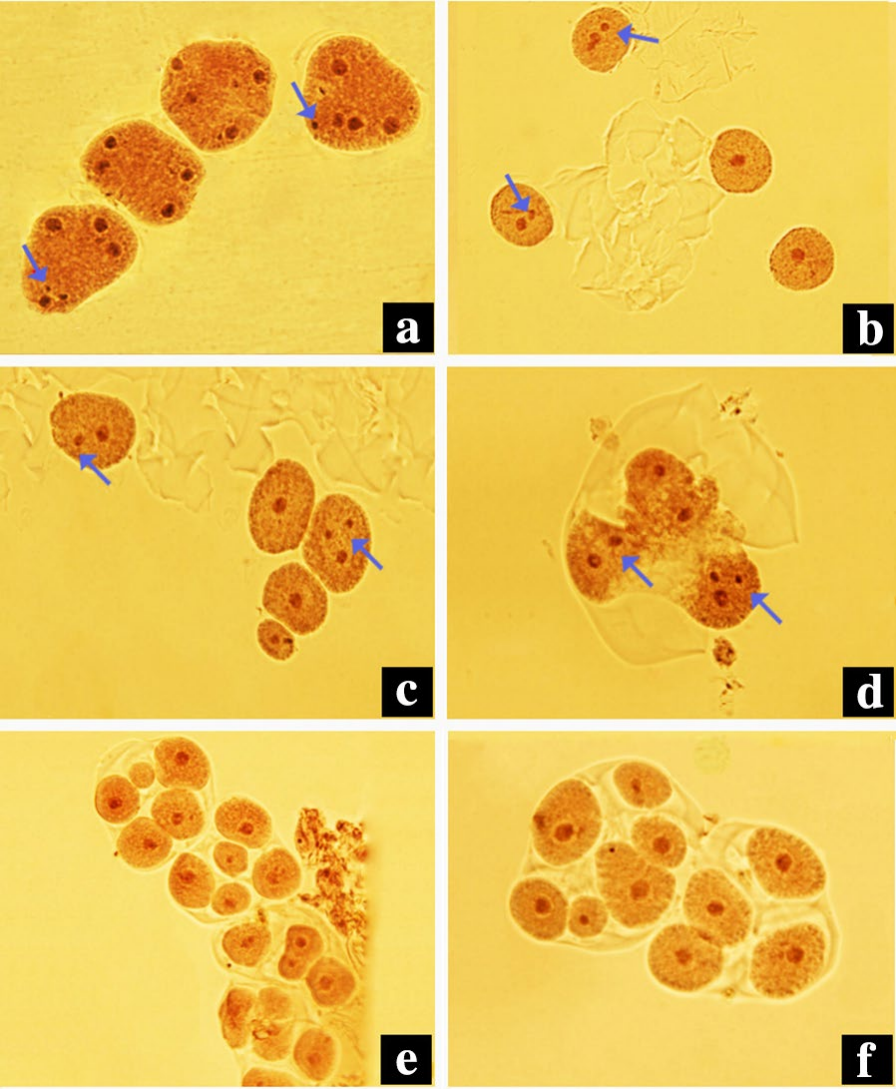
Abnormal cell division of PMC at meiotic TII stage of the induced tetraploid *F. tataricum* plants (**a** Tetrads with micronucleus; **b** tetrad with multiple nucleoli; **c** Pentad with multiple nucleoli; **d** triad with multiple nucleoli; **e** pentads; **f** hexads). *Arrows* show multiple nucleoli

Metaphase chromosome observation of root tip cells showed that the control plants are diploid with 8 pairs of chromosomes (2*n* = 2*x* = 16) (Fig. 1a–d). As expected, there are 16 chromosome pairs in the putative tetraploid (2*n* = 4*x* = 32) (Fig. 1e–h). Observations of the meiosis in PMCs confirmed the chromosome number of the putative tetraploid plants. At meiotic MI stage, chromosomes of diploid plants formed 8 bivalents (2*x* = 8II, Fig. 2c, d), while the putative tetraploid plants mostly formed 8 quadrivalents (4*x* = 8IV, Fig. 2a), occasionally 4 quadrivalents and 8 bivalents (4*x* = 4IV + 8II, Fig. 2b) were observed. At meiotic telophase II, some abnormal phenomenons, such as micronucleus (Fig. 3a–d), triads (Fig. 3d), pentads (Fig. 3e), and hexads (Fig. 3f) were observed in both of the control diploid and induced tetraploid plants, but the occurrence of abnormal phenomenons in the control diploid plants are obviously less than in those induced tetraploid plants (Table 2).

**Table 2:** Comparisons of meiotic TII stage between the induced tetraploids and their corresponding diploid *F. tataricum* cultivars

Cultivar	Tetraploid			Diploid			*t* ^a^	*t* ^b^
	Sum of cell	Abnormal cell frequency (%)	Micronucleus to cell ratio	Sum of cell	Abnormal cell frequency (%)	Micronucleus to cell ratio		
Qianwei 2#	132	30.30	0.5000	112	5.36	0.0536	4.9645**	7.6190**
Jinku 2#	107	25.23	0.4486	107	4.67	0.0467	4.2171**	6.8096**
Chuanqiao 1#	140	18.57	0.3857	159	4.40	0.0440	3.9015**	7.3160**
Liuqiao 1#	194	28.87	0.4897	124	4.03	0.0403	5.4857**	8.4178**
Average		25.05	0.4494		4.45	0.0445		

*t* ^a^ Indicate the *t* value of abnormal cell frequency (%)

*t* ^b^ Indicate the *t* value of micronucleus to cell ratio

** Indicate significance of the difference at the *p* = 0.01 level

### Morphological and physiological differences

**Fig. 4:**
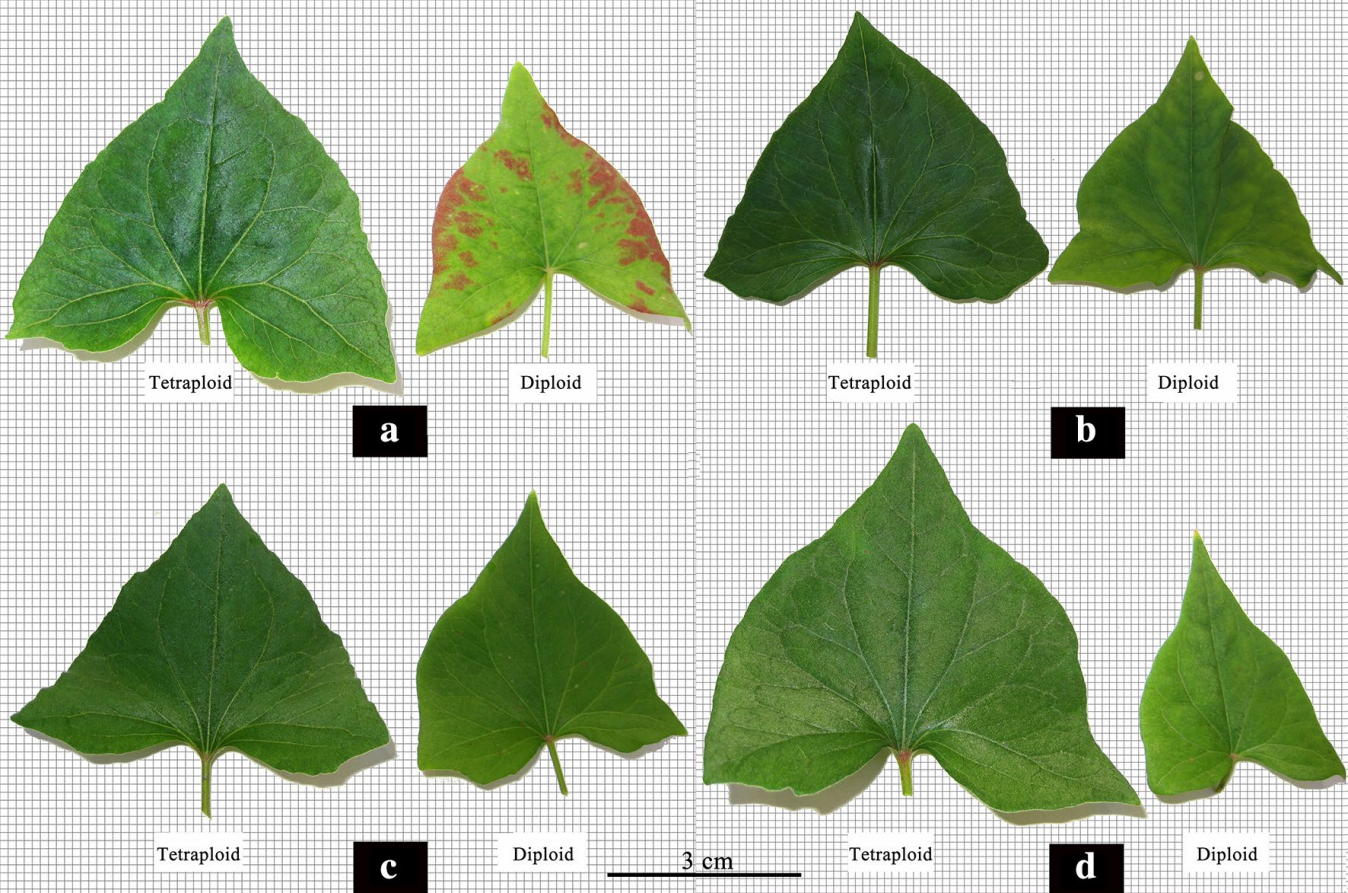
Leaves of the induced tetraploids and their corresponding diploid *F. tataricum* cultivars (**a** Qianwei 2#; **b** Jinku 2#; **c** Chuanqiao 1#; **d** Liuqiao 1#)

**Fig. 5:**
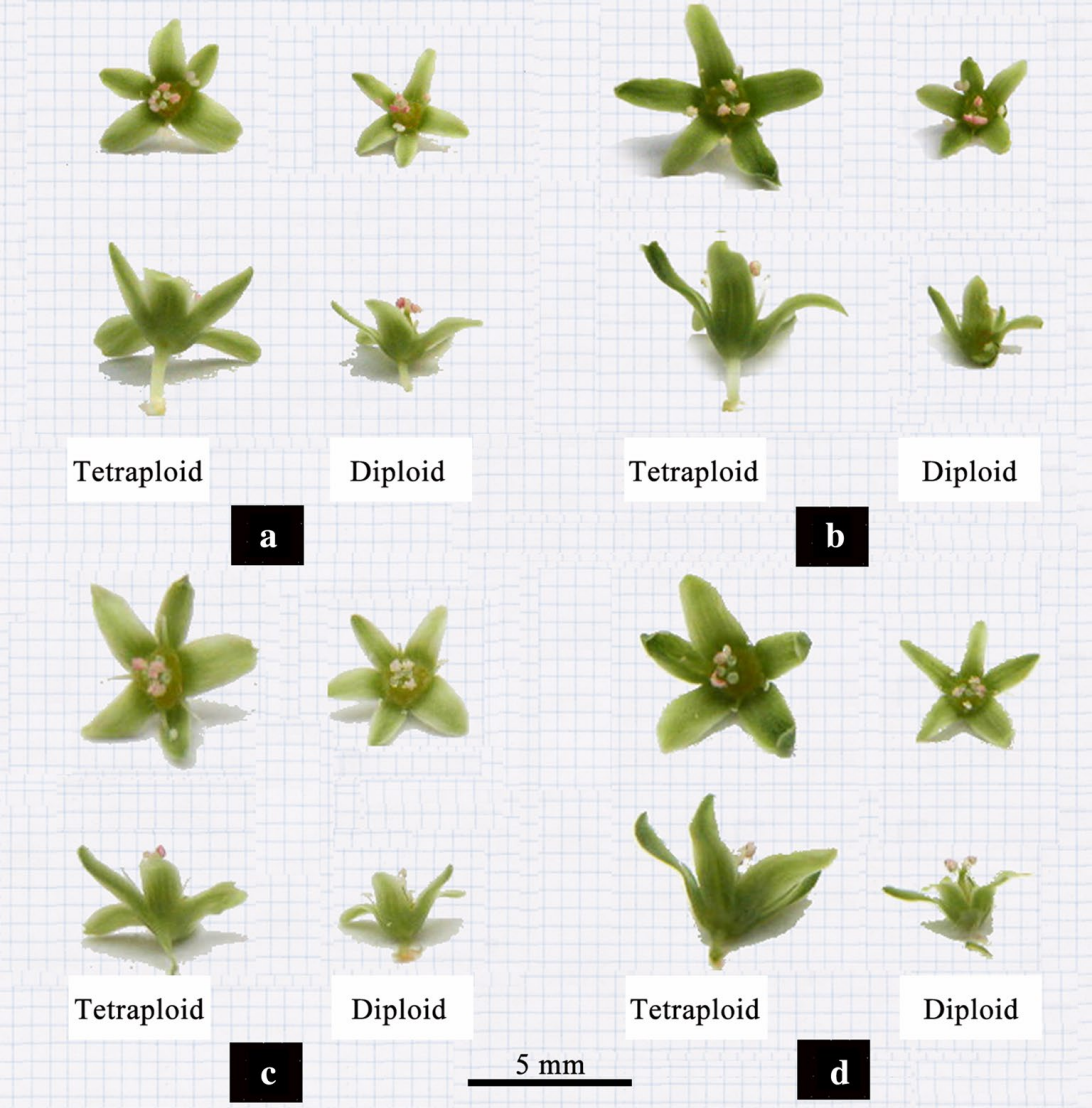
Flowers of the induced tetraploids and their corresponding diploid *F. tataricum* cultivars (**a** Qianwei 2#; **b** Jinku 2#; **c** Chuanqiao 1#; **d** Liuqiao 1#)

**Fig. 6:**
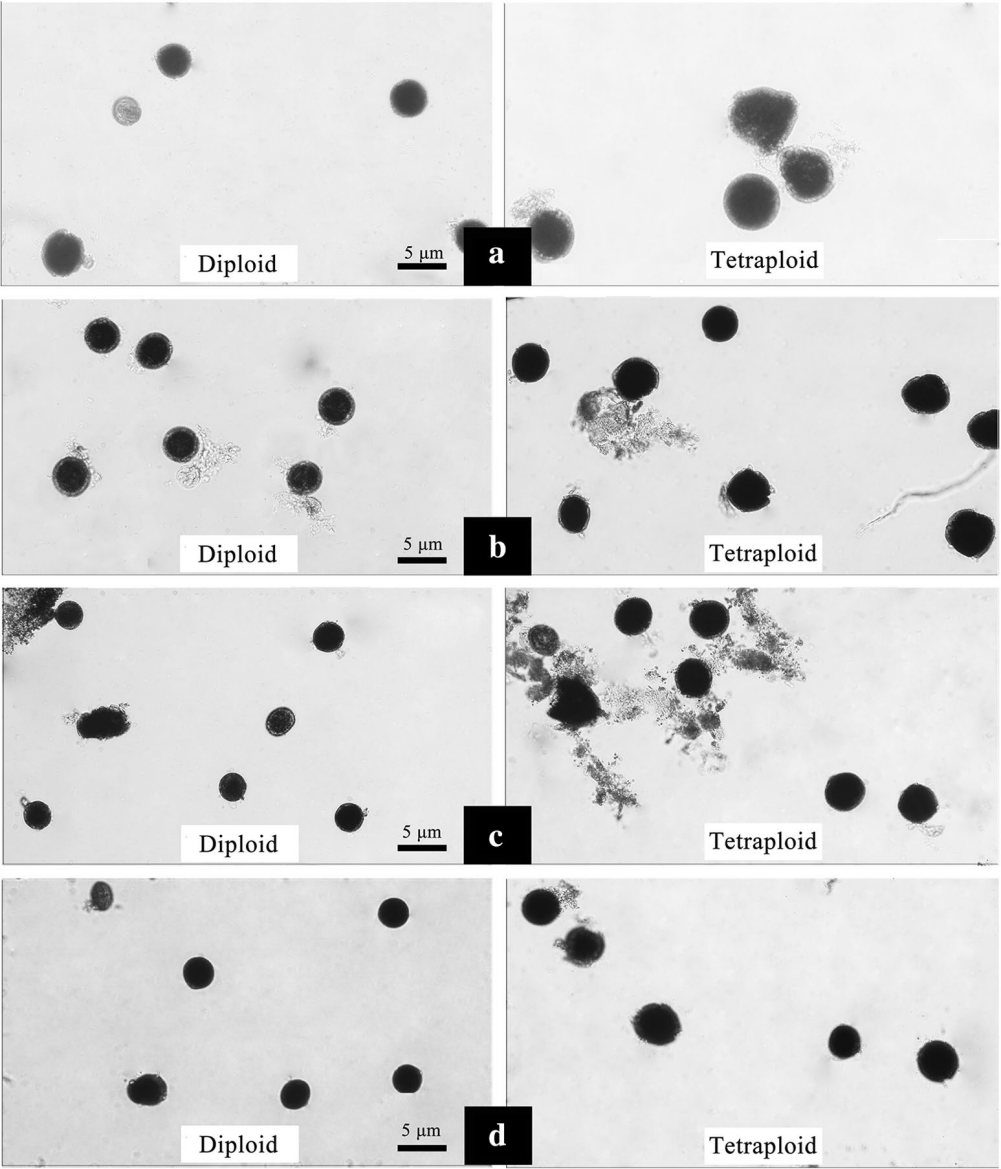
Pollen grains of the induced tetraploids and their corresponding diploid *F. tataricum* cultivars (**a** Qianwei 2#; **b** Jinku 2#; **c** Chuanqiao 1#; **d** Liuqiao 1#)

**Fig. 7:**
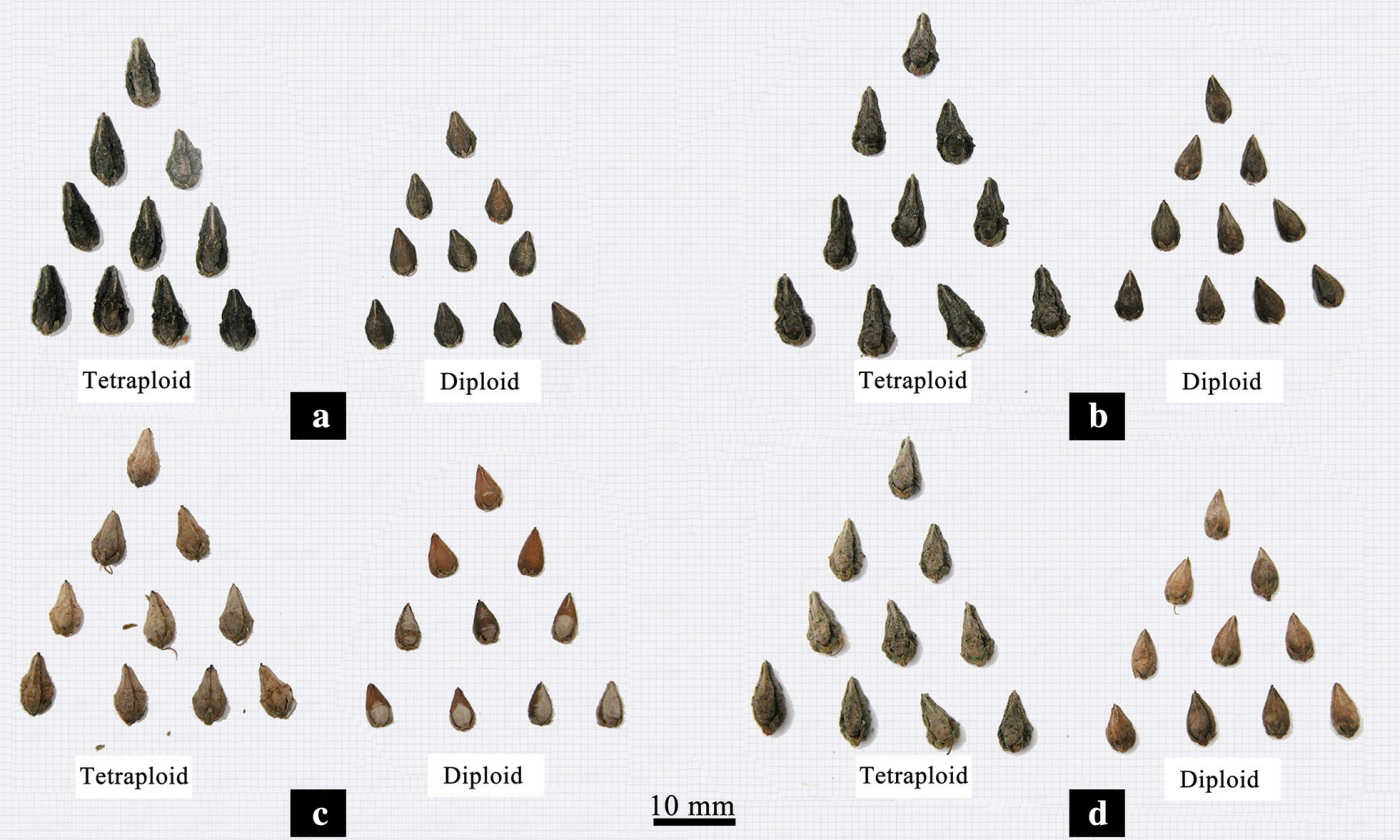
Seeds of the induced tetraploids and their corresponding diploid *F. tataricum* cultivars (**a** Qianwei 2#; **b** Jinku 2#; **c** Chuanqiao 1#; **d** Liuqiao 1#)

The comparison of vegetative characteristics and reproductive traits between the confirmed tetraploid plants and corresponding diploid plants of the four studied cultivars were presented in Tables 3 and 4, respectively. Results of two-sample *t* test statistical analysis indicated significant (*p* = 0.01) differences between the control diploid and induced tetraploid lines of *F. tataricum* with regard to the length, width and thickness of the leaves and the leaf index (Fig. 4). Compared with the corresponding diploid plants, the length, width and thickness of the leaves in all of the induced tetraploid plants had increased. The averages of increasing rate were 13.98, 37.40 and 139.33%, respectively. There were no significant differences (*p* = 0.05) in plant height, crown size or diameter of the basal stem between the control diploid and corresponding tetraploid lines in each of the four studied cultivars. However, regarding to transverse diameter of the flower bud, flower diameter, length of petal, and width of petal, significant differences (*p* = 0.01) were observed (Fig. 5). The diameters of pollen grains in the induced tetraploid plants were significantly larger (*p* = 0.01) than those in each corresponding diploid cultivars (Fig. 5), with an increasing rate of 28.13% in “Qianwei 2#”, 45.81% in “Jinku 3#”, 28.35% in “Chuanqiao 1#”, and 42.67% in “Liuqiao 1#” cultivar, respectively. Contrarily, the pollen vitality in the induced tetraploid plants were significantly lower (*p* = 0.05) than those in the corresponding diploid cultivars (Fig. 6). The seed size and thousand-seed weight in the induced tetraploid lines were obviously greater (*p* = 0.05) than those in the corresponding diploid cultivars (Fig. 7). Regarding to parameters of the average length of inflorescence, inflorescence number per individual, and floret number per inflorescence, no significant differences were observed between the control diploids and corresponding induced tetraploid plants.

**Table 3:** Comparisons of vegetative characteristics between the induced tetraploids and their corresponding diploid *F. tataricum* cultivars

Vegetative characteristic (cm)	Qianwei 2#		Jinku 2#		Chuanqiao 1#		Liuqiao 1#	
	4*x*	2*x*	4*x*	2*x*	4*x*	2*x*	4*x*	2*x*
Plant height	66.30 ± 20.20a	55.03 ± 5.35a	62.21 ± 15.34a	56.00 ± 10.23a	59.80 ± 21.02a	61.67 ± 2.34a	75.00 ± 23.12a	69.90 ± 11.20a
Plant breadth	40.23 ± 12.34a	45.89 ± 9.21a	39.36 ± 12.89a	51.24 ± 18.21a	45.34 ± 18.23a	43.89 ± 8.90a	53.34 ± 20.01a	50.34 ± 15.78a
Diameter of basal stem	1.03 ± 0.38a	0.98 ± 0.31a	1.11 ± 0.38a	1.02 ± 0.30a	0.99 ± 0.20a	0.96 ± 0.13a	1.20 ± 0.14a	1.21 ± 0.33a
Leaf length	6.12 ± 0.12A	5.28 ± 0.28B	6.39 ± 0.22A	5.63 ± 0.26B	6.56 ± 0.17A	5.86 ± 0.15B	7.23 ± 0.32A	6.31 ± 0.18B
Leaf width	4.02 ± 0.28A	3.34 ± 0.17B	4.21 ± 0.21A	3.25 ± 0.13B	3.99 ± 0.15A	3.05 ± 0.11B	5.32 ± 0.21A	3.15 ± 0.15B
Leaf thickness	0.95 ± 0.05A	0.38 ± 0.13B	0.83 ± 0.04A	0.35 ± 0.11A	0.88 ± 0.14A	0.41 ± 0.10B	0.92 ± 0.09A	0.36 ± 0.16B
Leaf index (length/width)	1.43 ± 0.22a	1.21 ± 0.25b	1.48 ± 0.31a	1.11 ± 0.32b	1.32 ± 0.18a	1.39 ± 0.20a	1.42 ± 0.23a	1.35 ± 0.19a

*Different small-case/capital letters* indicate the significance of difference between two mean values at the *p* = 0.05/0.01 level, respectively, as tested by two-sample *t* test, the same below

**Table 4:** Comparisons of reproductive characteristics between the induced tetraploids and their corresponding diploid *F. tataricum* cultivars

Reproductive characteristic	Qianwei 2#		Jinku 2#		Chuanqiao 1#		Liuqiao 1#	
	4*x*	2*x*	4*x*	2*x*	4*x*	2*x*	4*x*	2*x*
Vertical length of flower bud (mm)	4.12 ± 0.16A	3.23 ± 0.22B	4.01 ± 0.24a	3.36 ± 0.25b	3.99 ± 0.20a	3.37 ± 0.15b	4.19 ± 0.23A	3.30 ± 0.11B
Transverse diameter of flower bud (mm)	2.35 ± 0.18A	1.88 ± 0.21B	2.22 ± 0.12A	1.79 ± 0.20B	2.31 ± 0.15a	2.01 ± 0.11b	2.68 ± 0.17A	1.90 ± 0.23B
Flower diameter (mm)	5.15 ± 0.26A	3.68 ± 0.20B	6.35 ± 0.32A	3.24 ± 0.19B	5.45 ± 0.25A	3.56 ± 0.18B	6.41 ± 0.20A	3.29 ± 0.12B
Length of petal (mm)	2.69 ± 0.11A	1.63 ± 0.12B	3.01 ± 0.18A	1.51 ± 0.18B	2.23 ± 0.13A	1.25 ± 0.20B	2.95 ± 0.09A	1.17 ± 0.18B
Width of petal (mm)	0.90 ± 0.03A	0.52 ± 0.06B	0.97 ± 0.09A	0.49 ± 0.02B	0.88 ± 0.07A	0.40 ± 0.05B	0.95 ± 0.03A	0.38 ± 0.08B
Pollen grain diameter (μm)	5.01 ± 0.34A	3.91 ± 0.28B	5.57 ± 0.34A	3.82 ± 0.23B	4.98 ± 0.31A	3.88 ± 0.28B	5.55 ± 0.27A	3.89 ± 0.31B
Pollen-vigorous (%)	20.35 ± 1.38B	49.36 ± 3.21A	18.89 ± 1.34B	42.66 ± 2.89A	19.67 ± 1.89B	45.89 ± 2.92A	17.89 ± 1.25B	40.96 ± 3.01A
No. of inflorescences per plant	42 ± 6a	39 ± 8a	37 ± 7a	35 ± 8a	43 ± 7a	39 ± 5a	38 ± 9a	33 ± 5a
Average length of inflorescence (cm)	8.91 ± 4.25a	10.35 ± 4.36a	7.89 ± 5.02a	10.78 ± 5.21a	7.34 ± 4.56a	9.67 ± 3.89a	6.99 ± 4.23a	10.36 ± 5.01a
No. of flowers per inflorescence	34 ± 21a	67 ± 31a	42 ± 20a	73 ± 26a	39 ± 25a	71 ± 30a	49 ± 22a	77 ± 31a
Thousand-seed weight (g)	24.41 ± 3.67A	19.38 ± 2.25B	22.66 ± 1.89A	19.11 ± 1.12B	31.59 ± 1.35A	18.12 ± 0.98B	26.84 ± 2.01A	17.57 ± 1.03B
Seed size (mm)	8.29 ± 0.84A	5.86 ± 0.73B	8.10 ± 0.35A	6.01 ± 0.34B	7.20 ± 0.17A	5.63 ± 0.22B	7.17 ± 0.24A	5.42 ± 0.11B

*Different small-case/capital letters* indicate the significance of difference between two mean values at the *p* = 0.05/0.01 level, respectively, as tested by two-sample *t* test, the same below

In the process of plant cultivation, the resistance of the diploid and induced teteraploid lines to pathogens and insects were observed by the method of Mohammad et al. (2013). The main diseases and pests of *F. tataricum* in the area of Guizhou, China are powdery mildew, aphids and scale insects. Results showed that the resistance of the induced tetraploid lines to these pathogens and insects is stronger than those of the corresponding diploid plants. The average infection rate of the induced teraploid lines (7.93%, ranging from 3.1 to 10.5%) is significance less (*p* = 0.05) than those of the corresponding diploid plants (10.08%, ranging from 5.1 to 12.5%).

### Differences of seed protein and flavonoid content

The seed protein and flavonoid content of induced tetraploid and corresponding diploid lines of each of the four cultivars were determined and compared by the method of two-sample *t* test (Table 5). Results showed there are significant (*p* = 0.01) differences of seed protein and flavonoid content between the control diploid and corresponding tetraploid lines of *F. tataricum*. The seed protein content in the confirmed tetraploid plants were significantly higher (*p* = 0.01) than the corresponding diploid plants. The average increasing rates were: 12.75% in “Qianwei 2#”, 39.57% in “Jinku 2#”, 14.60% in “Chuanqiao 1#”, and 27.59% in “Liuqiao 1#” cultivar, respectively. Also, the seed flavonoid content in the tetraploid lines was significantly higher (*p* = 0.01) than the corresponding diploid lines, with an average increasing rates: 79.92% in “Qianwei 2#”, 28.86% in “Jinku 2#”, 10.10% in “Chuanqiao 1#”, and 51.15% in “Liuqiao 1#” cultivar, respectively.

**Table 5:** Comparisons of seed protein content and seed flavonoid content between induced tetraploids and their corresponding diploid *F. tataricum* cultivars

Content	Qianwei 2#		Jinku 2#		Chuanqiao 1#		Liuqiao 1#	
	4*x*	2*x*	4*x*	2*x*	4*x*	2*x*	4*x*	2*x*
Seed protein (%)	16.4 ± 0.32A	14.9 ± 0.45B	19.4 ± 1.56A	13.9 ± 1.14B	15.7 ± 1.14A	13.7 ± 1.03B	18.5 ± 1.21A	14.5 ± 0.96 B
Seed flavonoid (%)	4.57 ± 0.25 A	2.54 ± 0.18B	3.84 ± 0.38A	2.98 ± 0.27B	3.23 ± 0.22A	2.91 ± 0.16B	3.96 ± 0.31A	2.62 ± 0.11B

## Discussion

### Tetraploid induction and identification in *F. tataricum*

Polyploidization is a very important and valuable method for plant breeding to improve various important morphological and physiological characteristics and to generate valuable germplasm resources, and it has been successfully applied in many plant species (Tang et al. 2010; Dhooghe et al. 2011). Colchicine, as a chromosome doubling agent, has been widely applied to induce plant polyploidy because it disturbs the mitosis at the anaphase stage to result in chromosome doubling (Nigel et al. 2007; Pour et al. 2012). The tetraploid plants of common buckwheat were successfully induced with colchicines treatment by Zhu and Gao (1988) and Zhu et al. (1992); however, such research is very lacking in tartary buckwheat. In the present study, treatments with 0.25% (w/v) colchicines solution for three different durations of 48, 72, and 96 h were used to induce tetraploid plants in *F. tataricum*. From results, we conclude that the treated duration would obviously affect the efficiency of tetraploid induction and death rate of seedlings. Numerous previous studies indicated that the duration of colchicines treatment was positive correlation to the seedlings death rate in many plant species (Sikdar and Jolly 1994; Mohammad et al. 2013). Our study also showed the similar results in *F. tataricum*, indicating such effect may be common in plant. However, the duration of colchicines treatments which may cause higher death rate also resulted in higher efficiency to induce tetraploids (Chakraborti et al. 1998; Mohammad et al. 2013). Therefore, the suitable duration of the colchicines solution treatment is very important to obtain successful tetraploid induction. In the present study, the death rate of plants treated by colchicines ranging 2.0–10.0% was recorded. Ahloowalia (1967) reported that seedling mortality rate was 74.6% in *Lolium perenne* plants treated with 0.2% colchicines for 3 h, while Liu et al. (2007) reported that seedling mortality rate was 100% in *Platanus acerifolia* plants treated with 0.5% colchicines for 24 h. Due to a low mortality rate observed in the present study, we concluded that *F. tataricum* plants may have a high level of resistance to the colchicines toxicity. It can be also deduce that colchicines should be effective for producing tetraploids in other species of *Fagopyrum* genus because *F. tataricum* was effectively induced into tetraploids by colchicines.

Until now, several approaches have been used for the polyploidy identification in plants (Tandon and Bali 1957; Pei 1985; Li and Zhang 1991; Yang et al. 2006). Numerous early studies confirmed that the size and number of stomata and the number of chloroplasts within the guard cells of the induced tetraploid plant were significantly different from the corresponding diploid plants (Speckmann et al. 1965; Tan and Dunn 1973; Cohen and Yao 1996; Beck et al. 2003). In the event of chromosome doubling, morphological characteristics of plants can change significantly. Thus, morphological characteristics were useful markers in the identification of induced tetraploids. First, a large population of treated seedlings was pre-screened for putative tetraploid lines by morphological markers in the present study. Subsequently, the real tetraploid lines were confirmed by chromosome counting of root tip cells and meiosis in PMCs. This technological process is considered fast and effective for the identification of induced tetraploid plants. In the present study, the length, width, and thickness of leaves of the tetraploid plants induced from the four different *F. tataricum* cultivars significantly increased in comparison to the corresponding control diploid plants. Parameters of the flower diameter, seed size and weight of 1000 seeds also significantly increased in the induction tetraploid plants, in comparison to the control diploid plants. The chromosome number of *F. tataricum*, 2*n* = 2*x* = 16, was reported (Sheng et al. 2013). In the present study, as expected, the chromosome number of control diploid plants was 2*n* = 2*x* = 16 and the induced tetraploid plants 2*n* = 4*x* = 32 (Fig. 1). The autotetraploids, pre-screened by morphological markers, were confirmed by chromosome counting and PMCs meiosis observation. Results showed that applying the morphological characteristics as preliminary screening markers for the identification of induced tetraploid lines was feasible.

### Germplasm characteristics and utilization of tetraploid in *F. tataricum*

In the present study, all the four *F. tataricum* cultivars were successfully induced into tetraploid plants by colchicine. In comparison to the control diploid plants, several morphological characteristics were significantly increased in the induced tetraploid plants, such as leaf size, chlorophyll content, flower size and pollen diameter. Particularly, the important agronomic parameters of seeds, including seed size and thousand-seed weight, of the induced tetraploid plants were significantly greater than the control diploid plants. In addition, the seed protein and flavonoid content also significantly increases in the induced tetraploid plants. The improvement of morphology, physiology, and phytochemistry in the induced tetraploid lines obtain from the four *F. tataricum* cultivars suggest that tetraploids are useful materials for generating innovative germplasm resources in the genetics and breeding of genus *Fagopyrum*. Generally, polyploids have higher genetic adaptability and resistance to environmental stresses than diploids. Li et al. (2007) reported that induced tetraploids significantly increased their cold tolerance than corresponding diploid plants in the studied ornamental plants. While in the group of agricultural and medical plants, the induced tetraploids significantly increased biomass or effective compounds content. The present study also shows those induced tetraploid plants of *F. tataricum* display stronger resistance to environmental stress than corresponding diploids.

To further evaluate the potential value of induced tetraploids in the *F. tataricum* breeding, the meiosis in PMCs of the induced tetraploids and corresponding control diploids were analysed. The abnormities of meiosis in PMCs mainly happened at the MI and TII stag. The abnormal chromosome behaviours observed at MI and TII stages may imply the occurrence of disturbances in the process of meiosis at PMCs (Chen et al. 2004). In the present study, at MI and TII stage at the meiosis, a few of abnormal chromosomal behaviors and cell divisions, such as micronucleus, triads, pentads, and hexads, were observed in both of the control diploid and induced tetraploid plants, but the frequency of abnormities of the PMCs meiosis in induced tetraploid plants are significantly more than the control diploid plants. This result explains the low seed-setting percentage of the induced tetraploid lines. At MI stage of the meiosis of tetraploid plants, various chromosome configurations including bivalents, univalent, trivalent, and quadrivalent of chromosome synapsis were observed, showing that the meiotic chromosome synapsis of tetraploid plants is very complicated. This can be attributed to the chromosome doubling by colchicines treatment to form a autotetraploid which have four sets of each chromosome in the genome. The problems in chromosome synapsis among four homologous chromosomes of autotetraploid cause the unbalance of chromosome segregation and result in high genetic instability in pollen grains.

## Conclusions

*Fagopyrum tataricum* can be effectively induced into tetraploids by colchicines. In comparison to the control diploid plants, various agronomic traits may be significantly increased in the induced tetraploid plants, including seed size, thousand-seed weight, leaf size, chlorophyll content, flower size and pollen diameter. The content of seed protein and flavonoid may also be increased in the tetraploid plants. Moreover, the genetic instability in tetraploid plants also increased. The induced tetraploid materials can be used to generate innovative germplasm for the *Fagopyrum* species breeding.

## Abbreviations

PMCs: pollen mother cells
ANOVA: analysis of variance
